# Introduction to the Toxins Special Issue on Improved Analytical Technologies for the Detection of Natural Toxins and Their Metabolites in Food

**DOI:** 10.3390/toxins12080467

**Published:** 2020-07-22

**Authors:** Veronica Maria Teresa Lattanzio

**Affiliations:** Institute of Sciences of Food Production, National Research Council of Italy, Via Amendola, 122/O, 70126 Bari, Italy; veronica.lattanzio@ispa.cnr.it

Food, by nature, is a biological substrate and is therefore capable of supporting the growth of microbials that are potential producers of toxic compounds. Natural toxins include mycotoxins, marine biotoxins, plant toxins, cyanogenic glycosides, and toxins occurring in poisonous mushrooms. Natural toxins pose not only a risk to both human and animal health, but also impact food security and nutrition by reducing people’s access to healthy food. The tracking and detection of natural toxins in foods back to their source is a primary responsibility of food producers, distributors, handlers, and vendors. National authorities should conduct monitoring and ensure that levels of the most relevant natural toxins in food commodities comply with both national and international maximum levels or relevant toxicological thresholds.

This Special Issue of *Toxins* includes some recent advances in analytical methodologies for the detection of natural toxins in food commodities and biological fluids. The collected contributions are relevant to two main analytical approaches, namely advanced liquid chromatography–tandem mass spectrometry (LC-MS/MS) applications for the high sensitive and selective detection of emerging contaminants, and approaches for rapid and cost effective toxin detection based on immunoassays and cell-based assays.

The phytotherapeutic and nutritional use of plants and herbal-based products have increased in popularity over the last few years, however the improper usage of plants or unawareness of plant toxicity may lead to intoxication cases. Nowadays, highly sensitive and selective LC-MS/MS techniques represent a relatively easy to access and powerful tool to target plant toxins at low levels, and as a consequence, contaminants such as pyrrolizidine alkaloids and cardiac glycosides have surfaced as an issue of relevance in food safety. 

A method for the determination of pyrrolizidine alkaloids in honey has been set up by Hungerford et al. [1]. The study includes the results of a large survey on honey samples from Queensland (Australia), providing a picture of pyrrolizidine alkaloids incidence and levels and a possible correlation with their plant origin. Malysheva et al. proposed a new LC-MS/MS method for the quantification of cardiac glycosides in edible herbs and spices and, complementary to this, in human urine [2]. The results of the validation indicated that the developed method is able to detect and quantify the target cardiac glycosides at low levels making it suitable for application in food safety controls and analysis of human biological fluids to unravel the level of exposure to plant toxins.

Among the readily available in vitro methods, the functional assay known as the cell-based assay, which uses a neuroblastoma (N2a) cell line, is widely applied for routine surveillance of marine toxins. In their study, Viallon et al. optimized several key parameters of the cell-based assay, improving assay implementation and reliable toxin detection, making the final protocol suitable for future standardization and interlaborarory comparisons [3]. 

The overall performances of immunoassays strongly depend on the quality of selected immunoreagents, first of all, of the antibody. For this reason, the development and testing of new antibodies continues to warrant considerable efforts in current research. Maragos et al. proposed a new competitive enzyme-linked immunosorbent assay (ELISA) to screen for citreoviridin and its geometric isomer, iso-citreoviridin, in white rice based upon the isolation of two novel monoclonal antibodies [4]. The developed assays were relatively tolerant to methanol and acetonitrile and provided adequate analytical performances such as sensitivity, accuracy and precision, allowing for the screening of citreoviridin and iso-citreoviridin at levels that are toxicologically relevant. Studies by Bever et al. deal with the generation of novel monoclonal antibodies and their application in a competitive ELISA and a lateral flow immunoassay (LFIA) for the detection of amatoxins, lethal toxins found in a variety of mushroom species. The developed assays are intended for the screening of wild mushrooms (ELISA) and urine samples (LFIA), thus providing complementary tools for evaluating amatoxins occurrence and geographic distribution as well as directly determining amatoxin exposure of humans and animals [5,6].

When applying rapid screening tests for compliance testing, their fitness for purpose needs to be demonstrated through a validation study. The manuscript by Pecorelli et al. provides insights about the process of evaluating and comparing the performance profile of rapid methods currently applied for AFM1 screening in milk [7]. Addressing EU official guidelines, the analytical performances of the strip test and ELISA-based methods were evaluated, including verification of method performances through long-term quality control measurements and comparison with the AOAC reference method.

Antibodies as well as alternative receptors, such as aptamers, can be used in several biosensing platforms. The review by Zhuheng Li et al. updates the construction strategies of electrochemical biosensors such as immunosensors and aptasensors for the cost-effective determination of microbial toxins including bacterial toxins, fungal toxins and algal toxins [8]. The paper summarizes the roles of 2D nanomaterials and their nanocomposites in the configuration of electrochemical biosensors, while also discussing advantages, major challenges and perspectives of these electrochemical biosensors for future commercialization.

Overall, the papers included in this Special Issue have contributed to advance the state-of-art of analytical methods for the detection of natural toxins. Furthermore, a part of the published studies focused on emerging or less investigated toxins, thus providing the scientific community with new tools and/or data supporting a better understanding of related food safety issues.
